# Human Brain Microvascular Endothelial Cells and Umbilical Vein Endothelial Cells Differentially Facilitate Leukocyte Recruitment and Utilize Chemokines for T Cell Migration

**DOI:** 10.1155/2008/384982

**Published:** 2008-02-14

**Authors:** Shumei Man, Eroboghene E. Ubogu, Katherine A. Williams, Barbara Tucky, Melissa K. Callahan, Richard M. Ransohoff

**Affiliations:** Department of Neurosciences, Neuroinflammation Research Center, Lerner Research Institute, Cleveland Clinic, Cleveland, OH 44195, USA

## Abstract

Endothelial cells that functionally express blood brain barrier (BBB) properties are useful surrogates for studying leukocyte-endothelial cell interactions at the BBB. In this study, we compared two different endothelial cellular models: transfected human brain microvascular endothelial cells (THBMECs) and human umbilical vein endothelial cells (HUVECs). With each grow under optimal conditions, confluent THBMEC cultures showed continuous occludin and ZO-1 immunoreactivity, while HUVEC cultures exhibited punctate ZO-1 expression at sites of cell-cell contact only. Confluent THBMEC cultures on 24-well collagen-coated transwell inserts had significantly higher transendothelial electrical resistance (TEER) and lower solute permeability than HUVECs. Confluent THBMECs were more restrictive for mononuclear cell migration than HUVECs. Only THBMECs utilized abluminal CCL5 to facilitate T-lymphocyte migration *in vitro* although both THBMECs and HUVECs employed CCL3 to facilitate T cell migration. These data establish baseline conditions for using THBMECs to develop *in vitro* BBB models for studying leukocyte-endothelial interactions during neuroinflammation.

## 1. INTRODUCTION

Leukocyte recruitment across the blood brain barrier (BBB) into the perivascular space of the central nervous system (CNS) is a key step in the host defense response to pathogens, as well as in neurological disorders such as multiple sclerosis (MS), trauma, and stroke [1–3]. Static *in vitro* BBB models using brain microvascular endothelial cells (BMECs) have been used for studying the mechanism of leukocyte-endothelial interactions at the BBB [4–8]. Basic insights into leukocyte-endothelial interactions have been obtained through experiments using human umbilical vein endothelial cells (HUVECs) in both static and dynamic conditions [9–11]. Because endothelial cells from different vascular beds are uniquely adapted to meet the demands of the underlying tissues [12, 13], data from studying HUVECs may not be directly applicable to leukocyte-endothelial interactions at the BBB. In this regard, differences between BMECs and HUVECs have been reported [14–17]. Transfected human brain microvascular endothelial cells (THBMECs) were isolated from human brain microvessels and immortalized by transfection with simian virus 40 large T antigen (SV40-LT) [14]. THBMECs share characteristics of primary human brain microvascular endothelial cells (HBMECs) including expression of tight junction (TJ)-associated proteins and high transendothelial electrical resistance (TEER) [4, 14]. THBMECs express factor VIII-related antigen and gamma-glutamyl transpeptidase, and take up $1,1^{\prime }$-dioctadecyl-$3,3,3,3^{\prime }$-tetramethylindocarboxyamine perchlorate-labeled acetylated low-density lipoprotein [18]. In this paper, we compare selected features of THBMECs and HUVECs and focus on their capacity for utilizing abluminal endothelial cell derived chemokines to facilitate mononuclear cell transmigration *in vitro*.

Chemokines have been proposed to play a major role in the activation and recruitment of leukocytes to sites of inflammation. CCL5 is a chemoattractant for multiple leukocyte subtypes (predominantly T cells) via three known G protein-coupled receptors (GPCRs), CCR1, CCR3, and CCR5 [19] while CCL3 signals towards a similar spectrum of mononuclear cells. CCL3 and CCL5 are produced in the CNS of individuals with several neuroinflammatory diseases including MS. Using confluent THBMEC culture as an *in vitro* BBB model, anti-CCR1 and anti-CCR5 antibodies completely abrogated CCL5-driven mononuclear cell migration across a cytokine-activated BBB [5]. The data in this paper showed that THBMECs differed from HUVECs in their ability to use abluminal CCL5 to mediate T cell migration. These results characterize specific features that distinguish THBMEC from HUVEC cultures, and will help clarify conditions for the development of models to study leukocyte transmigration across BBB *in vitro*.

## 2. METHODS

### 2.1. Endothelial cells culture and leukocyte preparation

THBMECs are adult human brain microvascular endothelial cells transfected and immortalized with a plasmid containing SV40-LT [4–6]. THBMECs were grown in RPMI 1640 containing 10% heat-inactivated fetal bovine serum, 10% Nu-Serum, 2 mM L-glutamine, 1 mM pyruvate, essential amino acids, and vitamins. The HUVEC cell lines were purchased from American Type Culture Collection ATCC (ATCC Number CRL-1730™) and cultured in Ham's F12K medium adjusted to contain 2 mM L-glutamine, 1.5 g/L sodium bicarbonate, 0.1 mg/mL heparin, 0.03 mg/mL endothelial cell growth supplement (ECGS), and 10% fetal bovine serum. The THBMECs used in this paper were passages 19–25 and HUVECs were passages 3–7. In order to address their basic characters, endothelial cells in this paper were not stimulated with cytokines. Peripheral blood mononuclear cells (PBMCs) were isolated from fresh whole heparinized blood of healthy volunteers by density centrifugation using lymphocyte separation medium (Mediatech Inc., Herndon, VA) as previously described [4–6]. PBMCs were resuspended at 10^7^ cells/mL in transendothelial migration (TEM) buffer (RPMI 1640 without phenol red +1% bovine serum albumin) for transmigration assays. For quantification of transmigrated cells, PBMCs were labeled with calcein-AM (Molecular Probes Inc., Eugene, OR) according to the manufacturer's instructions and resuspended in the original volume of TEM buffer prior to transmigration assays. The research protocol was approved by the local institutional review board and signed informed consent was obtained from all donors studied.

### 2.2. Transendothelial electrical resistance (TEER) and solute permeability

THBMECs and HUVECs were cultured to confluence on 24-well collagen-coated Transwell^TM^ tissue culture inserts (Corning Costar Inc., Corning, NY). TEER and solute permeability were measured as previously described [4–6, 18]. TEER was measured using an EVOM voltohmmeter (World Precision Instruments Inc., Sarasota, FL). Solute permeability was assessed using fluoresceinated dextran-70 (1 mg/ml, Sigma Inc., St. Louis) and fluorescent recovery in the lower chamber was measured after 15 minutes using a SPECTRAmax GEMINIXS microplate spectrafluorometer (Molecular Devices Corp., Sunnyvale, CA). Solute permeability was calculated using the following formula: (lower chamber fluorescence/input fluorescence) × 100%. TEER and solute permeability was determined in triplicate.

### 2.3. Immunocytochemistry

ZO-1 and occludin were detected by indirect immunocytochemistry on confluent THBMECs and HUVECs as previously described [20]. Polyclonal rabbit anti-human ZO-1 and anti-human occludin antibodies (1:100; Zymed Laboratories Inc., San Francisco, CA) were used in combination with mouse antirabbit IgG conjugated to FITC (1:100; Southern Biotechnology Associates Inc., Birmingham, AL). Slides were viewed using a Leica Aristoplan laser scanning confocal microscope (Leica Wetzlar, Heidelberg , Germany).

### 2.4. Transmigration assays

Transmigration assays were performed with Transwell ^TM^ inserts containing confluent THBMEC or HUVEC culture. Unlabeled and calcein-AM-labeled PBMCs were utilized in parallel for the quantification of PBMC transmigration and subpopuation analyses as previously described [4–6]. 10^6^ PBMCs from the same donor suspended in TEM buffer were added to the insert and allowed to transmigrate at 37°C in a 100% humid atmosphere with 5% CO_2_ for the indicated time. For chemokine-driven migration, 600 *μ*L of TEM buffer containing 50 ng/mL CCL3 or 100 ng/mL CCL5 was placed in the lower chamber (abluminal side of the endothelium). These concentrations had been previously shown to maximally induce PBMCs migration in transmigration assays (data not shown). Migrated cells were collected for subpopulation analysis using flow cytometry. The migration ratio for each leukocyte subpopulation was calculated with the following formula: [total number of migrated calcein-AM-labeled PBMCs × subpopulation percentage in migrated PBMCs]/[total number of input PBMCs × subpopulation percentage in input PBMCs] × 100%. These assays were performed in quadruplicate, with migration without added abluminal chemokine serving as controls.

### 2.5. Antibody staining and flow cytometry

Four wells containing migrated PBMCs were pooled for flow cytometric staining using anti-CD3 PerCP (clone SK7) and anti-CD14 APC (Leu-3A, all from Becton Dickinson Biosciences, San Jose, CA) as previously described [4–6] Data were collected using an LSR flow cytometer (BD Immunocytometry Systems, San Jose , CA). Analysis was performed using FlowJo® software (Tree Star, Ashland Inc., OR). Monocytes and lymphocytes were gated according to forward and side scatter, as well as CD3 and CD14 staining profiles and analyzed against isotype-matched controls.

### 2.6. Data analysis

Statistically significant differences between groups were determined by Student's t-test, and values of P<.05 were considered significant.

## 3. RESULTS

### 3.1. Different patterns of occludin and ZO-1 immunoreactivity in confluent THBMECs and HUVECs

**Figure 1:**
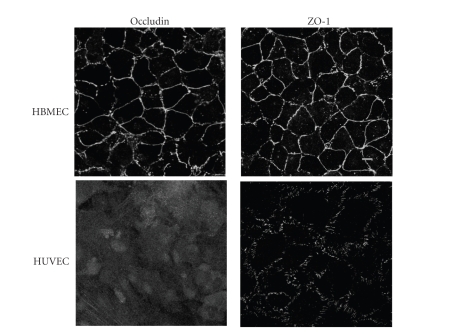
*Confluent THBMEC cultures possess intercellular TJs while HUVECs do not.* Three different passages of THBMECs and HUVECs were cultured to confluence. Endothelial cells were stained with rabbit anti-human ZO-1 and occludin antibodies followed by FITC conjugated goat anti-rabbit IgG. Digital pictures were taken following visualization with a Leica Aristoplan laser scanning confocal microscope. THBMEC cultures exhibited continuous ZO-1 and occludin expression at sites of intercellular contact. In contrast, HUVEC cultures showed punctate ZO-1 staining and no occludin immunoreactivity. Scale bar 10 *μ*m.

HUVEC have been reported to express TJ proteins when cultured with human astrocyte-conditioned medium [21]. We addressed TJprotein expression by HUVECs in tissue culture conditions that included ECGS with bovine pituitary extract. Occludin and ZO-1 have regulatory and signaling functions, acting as cytoskeletal linkers interacting with elements of the actin cytoskeleton [22]. We assessed ZO-1 and occludin expression by fluorescent immunocytochemistry using 3 different passages of THBMECs and HUVECs. THBMECs exhibited strong and continuous ZO-1 and occludin expression at sites of intercellular contact. In contrast, HUVECs showed no occludin expression and discontinuous, punctuate ZO-1 staining at the cell-cell interfaces (see Figure 1). We stained the TJ associated proteins on HUVECs from passages 3–7 and THBMECs of passages 19–25 and obtained consistent expression patterns.

### 3.2. Confluent THBMEC cultures have a higher TEER and lower solute permeability than HUVEC

**Figure 2:**
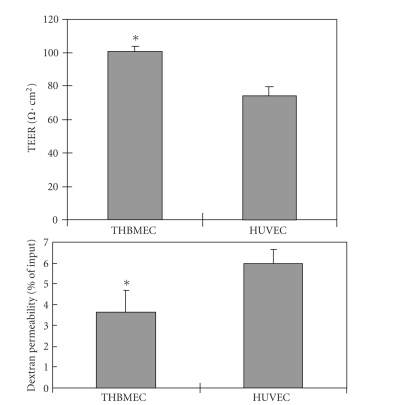
*Confluent THBMEC cultures have a higher TEER and lower solute permeability than HUVEC.* THBMECs and HUVECs were cultured to confluence on 24-well collagen-coated Transwell^TM^ inserts. TEER (a) was measure using an EVOM voltohmmeter. THBMECs possess higher TEER values. Solute permeability (b) was assessed by adding 100 *μ*l of 1 mg/mL fluoresceinated dextran-70 in Transwell^TM^ inserts. Fluorescent recovery in the lower chamber (well) was measured after 15 minutes as stated in Section 2. Triplicate wells were measured and inserts without cultured endothelial cells were used as controls (data not shown). THBMEC cultures demonstrate a lower solute permeability. *indicates P<.05 compared with HUVECs.

**Figure 3:**
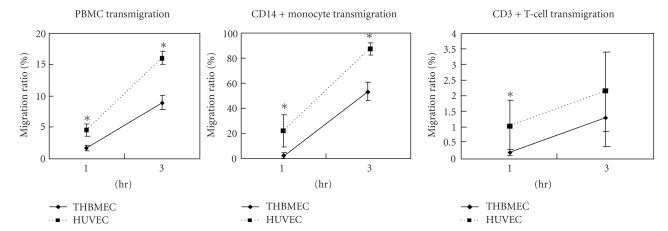
*THBMEC cultures are more restrictive towards PBMCs transmigration than HUVECs.* 10^6^ of PBMCs were added into the transwell inserts containing confluent THBMECs or HUVECs and allowed to transmigrate for the indicated time. Migrated cells were collected and three color stainings were performed in one step using anti-CD3 PerCP and anti-CD14 APC antibodies. Data were collected with an LSR flow cytometer and analyzed using FlowJo® software. Parallel migration assays were performed using calcein-AM labeled and unlabeled PBMCs, and migration ratios calculated as stated in Section 2. The assays were performed in quadruplicate using four different donors. Confluent HUVEC cultures are more permissive towards PBMCs migration than THBMECs. *indicates P<.05 comparing THBMECs with HUVECs. PBMCs transmigration across these endothelial cultures follows the pattern: monocytes > T cells > B cells (data about B cells not shown).

TEER and solute permeability are often used to evaluate the physical properties of endothelial intercellular junctions [4, 5, 23]. Confluent THBMECs exhibited mean TEER of 100 Ω⋅cm^2^, values that are consistent with a previous report for primary brain microvascular endothelial cells [24] while HUVECs attained an average TEER of 74 Ω⋅cm^2^ at passage 3 (see Figure 3), which decreased to 33 Ω⋅cm^2^ at passages 5–7 (data not shown). Confluent THBMECs consistently exhibited a low solute permeability, with average fluorescence recovery in bottom wells of 3.4% of input, compared to 6% for HUVECs at passage 3 (see Figure 2). Solute permeability of HUVECs increased to 15.9% at passages 5–7. These data demonstrated that some barrier properties of HUVECs were passage dependent.

### 3.3. THBMECs are more restrictive towards PBMC transmigration than HUVECs

We initially determined the kinetics of PBMCs migration across THBMEC and HUVEC cultures. The migration of calcein-AM labeled PBMCs across THBMECs and HUVECs, as a percentage of input cells, progressively increased over time (see Figure 3). At passage 3, HUVECs were more permissive than THBMECs for PBMCs migration at both 1 hour and 3 hours (as in Figure 3). The findings are consistent with those previously repeated for passages 5–7 HUVEC. We also ascertained the kinetics of PBMCs subpopulation migration. There was a progressive increase in monocyte and T lymphocyte migration across both THBMEC and HUVEC cultures, with higher numbers observed at all time points with confluent HUVECs (Figure 3). This observation demonstrated that confluent THBMEC cultures were more restrictive towards the migration of both monocytes and T cells than HUVEC.

### 3.4. THBMEC cultures utilize both CCL3 and CCL5 to drive T cell migration while HUVEC utilize only CCL3

Chemokines are produced primarily by astrocytes, microglia endothelial cells, and infiltrating leukocytes during neuroinflammatory disorders such as MS. It remains uncertain how chemokines produced in the brain parenchyma or within endothelial cells can be secreted, transported, and immobilized, so as to signal to circulating leukocytes. In order to study question *in vitro*, a BBB model capable of utilizing those chemokines highly expressed in neuroinflammatory lesions would be advantageous.

**Figure 4:**
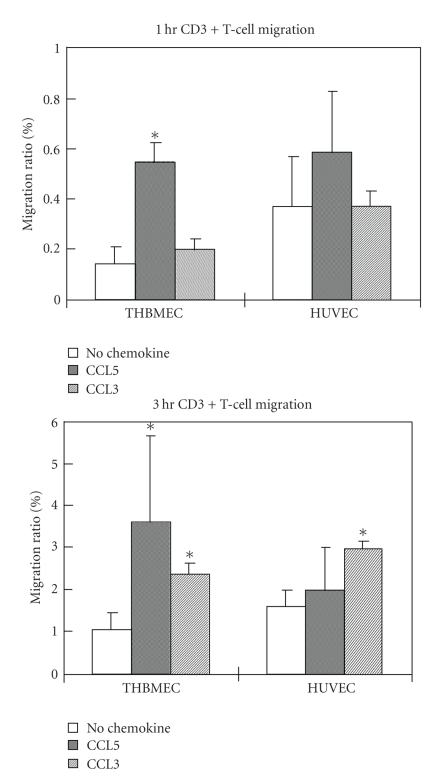
*THBMEC cultures utilize both CCL3 and CCL5 to drive T cell migration while HUVEC cultures utilize CCL3 only.* THBMECs or HUVECs were cultured to confluence on Transwell ^TM^ inserts. 10^6^ of PBMCs from the same donor were added into the inserts with or without 50 ng/mL CCL3 or 100 ng/mL CCL5 added to the lower chamber (well). Migration ratios were calculated as described in Section 2. CCL3 facilitated T cell migration across both THBMECs and HUVECs while CCL5 induced T cell migration across THBMECs only. The assays were performed in quadruplicate for each donor and three donors were included. *indicates *P* < .05 compared to basal migration without added chemokine.

Transmigration assays were performed with CCL3 or CCL5 introduced in the abluminal side of the Transwell^TM^ system. CCL5 induced an approximately 5-fold increase in CD3+ T cell migration across THBMECs relative to basal migration at 1 hour and 3-fold increase at 3 hours (P<.05). In contrast, adding CCL5 to the bottom chamber did not induce a significant change in CD3+ T cell migration across HUVECs (Figure 4). In contrast to CCL5, CCL3 increased CD3+ T cell migration across both THBMECs and HUVECs at 3 hours (Figure 4). We also compared CCL5 driven migration in cultured THBMECs and HUVECs at passage 3 and found that lower passage HUVECs did not utilize abluminal CCL5 to facilitate T cell migration (data not shown).

## 4. DISCUSSION

We compared selected morphological and functional features of THBMEC and HUVEC cultures. This analysis included junctional protein expression, restriction and selection in leukocyte recruitment, and utilization of abluminal CCL3 or CCL5 to drive T cell migration. Our data continue and extend prior findings from J.S. Pachtor and his colleagues (Andjelkovic, IVCDB, 2001). This group developed a novel model to visualize CCL2-driven monocyte transendothelial migration into a subendothelial collagen gel. Compatible with the current report, Pachter et al. found that BMECs were less permissive than HUVECs for monocyte transendothelial migration. Our study utilized different methods, and also added the new information that T cell migration is equally affected. Further, we found that CCL5 is selectively used by THBMECs but not by HUVECs to promote T cell transmigration. The data showed that morphological and functional differences exist between THBMECs and HUVECs under the defined culture conditions used in these studies. The findings were consistent with prior reports [14–17]. It has been reported that BMECs but not HUVECs express genes that are important in immunoregulation (OSM-R beta, decorin, IL-6), growth support (brain-derived neurotrophic factor, stem cell factor, transforming growth factor-beta), and angiogenesis (VEGF, erbB1) [25]. In addition, differential expression of adhesion molecules between THBMECs and HUVECs has been described [14]. Our data focus on functional differences between THBMEC and HUVEC cells with regard to PBMCs transmigration *in vitro*.

The structural peculiarities of endothelial cells from different tissues or organs are adapted to meet the demands of the underlying tissue. Intercellular TJs play a key role in maintaining homeostasis of the brain. The heteropolymers of occludin and claudin form the intramembrane strands of TJs while ZO-1 is involved in the formation of cytoplasmic plaques that connect with occludin [26]. Occludin and ZO-1 also function as cytoskeletal linkers interacting with actin cytoskeleton, and are involved in TJ assembly signaling pathways [22]. Strong occludin expression is unique to cerebral endothelial cells and plays a crucial role in the control of vascular permeability, since tissue expression of occludin correlates well with barrier properties [27]. Although we did not address the ultrastructural presence of tight junctions, our experiments showed that confluent THBMEC cultures exhibited continuous occludin and ZO-1 immunoreactivity at points of intercellular contact while confluent HUVECs (passages 3–7) exhibited a lack of occludin and punctate ZO-1 expression. It has been reported that HUVECs express occludin and ZO-1 upon culture with astrocyte conditioned medium [11], and that primary HUVECs expressed occludin at intercellular junctions in BioWhittaker's endothelial cell growth medium, which contains bovine brain extract [28]. Given these disparate findings, it is likely that passage and culture conditions greatly affect the intercellular TJ protein expression in HUVECs while THBMECs consistently express intercellular TJ components. It should be noted that culture conditions for HUVECs and THBMECs differ, and these differences may have contributed to the results we report here.

The interaction between circulating leukocytes and the endothelium of BBB is a crucial step in diverse pathologic processes [29, 30]. THBMEC cultures, from passage 19 to 25, in contrast with HUVEC cultures from passage 3 to 7, were extremely restrictive towards PBMCs transmigration. Interestingly, although HUVECs at earlier passage showed higher TEER and lower solute permeability than higher passages, they supported more PBMCs transmigration than THBMECs. Currently, there are mainly three potential routes known for leukocyte diapedesis across endothelial cells: paracellular, transcellular, and tricellular corners [11, 31, 32]. Our data do not address whether there is increased PBMCs paracellular migration across HUVECs.

In our *in vitro* transmigration assays, monocytes had much higher migration efficiency across both THBMEC and HUVEC cultures than lymphocytes. About 50 percent of input CD14+ monocytes migrated across confluent THBMEC cultures after 3 hours, while more than 85 percent of input CD14+ monocytes migrated across HUVEC cultures at this time point. Although the current studies do not address this, it is possible that the expression of specific chemokine receptors, leukointegrins, and adhesion molecules facilitates higher rates of monocyte migration in these assays. The CD14+ monocyte subset comprises nearly 90% of peripheral blood monocytes and the vast majority of these cells express CCR1, CCR2, CXCR2, CXCR4, and *α*_4_ *β*_1_ integrins [14, 33–36]. Our previous work showed that most input CD14+ monocytes express CCR2 and THBMECs produce CCL2 under resting conditions [4–6]. We also demonstrated that confluent THBMECs express fibronectin connecting segment-1 (FN CS-1), a high affinity receptor for *α*_4_ *β*_1_ integrin, and showed that chemokine-driven CD14+ monocyte transmigration was dependent on *α*_4_ *β*_1_/FN CS-1 interactions [5]. These observations suggest that CCR2+ CD14+ monocytes interact with endogenous CCL2 produced by THBMECs, with resultant chemokine-induced *α*_4_ *β*_1_ integrin activation, followed by transmigration through *α*_4_ *β*_1_ integrin/FN CS-1 interactions. Analogous mechanisms may explain the high rates of CD14+ monocyte migration across HUVECs.

T cells migrated at much lower rates, compared to monocytes in these assays, across both THBMECs and HUVECs. It is worth noting that activated lymphocytes, which migrate efficiently in these assays, are present at relatively low abundance (10–20% of PBMCs) in the circulation. The low rates of T cell migration could also be explained partially by the observation that HUVECs and THBMECs exhibit low constitutive expression of ICAM-1, an important determinant for T cell adhesion and transmigration [5, 37, 38]. The specific expression of chemokines and adhesion molecules doubtless plays key roles in the selective recruitment of leukocytes across the BBB [33, 34].

Finally, in this study, we addressed the capability of THBMECs and HUVECs to utilize chemokines CCL3 and CCL5 to enhance PBMCs transmigration *in vitro*. Unexpectedly, the data showed that confluent THBMECs could utilize both CCL3 and CCL5 to facilitate T cell migration, while HUVECs only employed CCL3 to drive T cell migration *in vitro*. Our data do not address the mechanisms underlying these differences. Characterizing the properties of peripherally-derived and CNS-derived endothelial cells in vitro will aid interpretation of data from model systems in the context of inflammatory CNS disease.
